# Age-related changes of microbiota in midlife associated with reduced saccharolytic potential: an in vitro study

**DOI:** 10.1186/s12866-021-02103-7

**Published:** 2021-02-15

**Authors:** Junkui Chen, Xionge Pi, Wei Liu, Qunfang Ding, Xin Wang, Weiguo Jia, Liying Zhu

**Affiliations:** 1 State Key Laboratory for Managing Biotic and Chemical Threats to the Quality and Safety of Agro-products, Institute of Plant Protection and Microbiology, Zhejiang Academy of Agricultural Sciences, Hangzhou, P. R. China; 2 The Center of Gerontology and Geriatrics, National Clinical Research Center of Geriatrics, West China Hospital, Sichuan University, Chengdu, P. R. China

**Keywords:** Midlife, Microbiota, In vitro fermentation, SCFAs, Acetate, Gas production

## Abstract

**Background:**

Gut microbiota is critical in maintaining human health, of which diversity and abundance are subject to significantly reduce in seniors. Gut microbiota is reported to be stable across the long adulthood in general, but lack of careful examination, especially for the midlife people.

**Results:**

To characterize the gut microbiota in midlife, we investigated the faecal microbiota between two groups of healthy people, young, 20–39 years old, *n* = 15; and midlife, 40–60 years old, *n* = 15. Metabolic responses of the microbiota were studied through in vitro batch fermentation model. Although no difference was observed in the diversity indices between the two age groups, a wide range taxonomic changes were found in the faecal microbiota. Furthermore, substantial *Bifidobacterium* reduction was also found in both faecal and fermented samples. The faecal SCFAs are similar in both groups, as well as starch fermentation broth. However, after inulin fermentation, the acetate concentration and inulin degradation rate decreased while the gas production increased in midlife group, suggesting a deficiency of saccharolytic potential in midlife, especially for non-digestible carbohydrate.

**Conclusions:**

Our data demonstrate that gut microbiota begins to change as early as in midlife. The reduction in *Bifidobacterium* dominates the change of the microbiota composition in midlife resulting in attenuated saccharolytic capacity of inulin, possibly leading to insufficient acetate production which might be associated with healthy problems in this transition period from young to elderly.

**Supplementary Information:**

The online version contains supplementary material available at 10.1186/s12866-021-02103-7.

## Background

As human life expectancy increases, aging has become a public health issue. Numerous studies demonstrate that gut microbiota plays key roles in aging process [1–4]. Compared with young adults, the gut microbiota of elderly lacks diversity and is less stable combined with increased abundance of Bacteroidetes, Proteobacteria, particularly Gammaproteobacteria, and decreased abundance of Firmicutes and bifidobacteria [3]. The aging-related dysbiosis of gut microbiota not only causes intestinal disorders but also extra-intestinal diseases, such as reduction of innate immunity, sarcopenia, and cognitive decline [5].

Midlife is a transition between young adulthood and elderly, when many physiological and psychological changes occur [6], leading to high morbidity rate, such as obesity [7] and type 2 diabetes (T2D) [8]. Furthermore, overweight/obesity and the vascular risk factors in midlife are demonstrated to associate with the later development of dementia [9, 10]. One interesting question is whether gut microbiota starts to change in healthy midlife people. Previous mice study of three age groups has shown that polysaccharide utilization is higher in middle-aged mice than older mice [11]. An integrative study of aging mice demonstrated that the relative abundance of *Akkermansia* spp*.*, *Bifidobacterium* spp*.* and *Lactobacillus* spp*.* decreases at middle age compared to young subjects [12]. Recently, Boehme and colleagues revealed a strong basal and stress-induced neuro inflammatory profile in middle-aged mice, which is associated with the changes in both microbiota compositions and their metabolites [6]. Interestingly, transfer of gut microbiota from young to middle-aged subjects extended the lifespan of the short-lived killifish [13]. Studies of microbiota in midlife human are highly limited. A recent cohort study of middle age (50–59 years old) to elderly individuals (> 60 years old) revealed that the presence of Bacteroidetes is positively associated with CD8^+^ and negatively with CD4^+^/CD8^+^ ratio, indicating tendency to immune diseases such as chronic infection, cancer or HIV [14].

Short chain fatty acids (SCFAs) are the primary metabolites produced by bacterial fermentation of non-digestible carbohydrates in the gastrointestinal tract. SCFAs act as important molecular signals between the microbiota and host [15], and regulate glucose homeostasis, appetite, energy intake, neuronal activity, immune system and inflammatory response [16]. In metabolic diseases, such as obesity, T2D [17, 18], cardiovascular diseases [19], as well as psychological dysfunction including cognitive process [20], SCFAs are regarded to play central roles. Based on the metagenomic studies, the gut microbiota of elderly is characterized by loss of genes involved in the SCFAs synthesis and decrease in the saccharolytic potential [21]. Six individual SCFAs, especially acetate, were reported to decrease in elderly compared to young subjects in a study of Parkinson’s disease [22]. But SCFAs profile in midlife human is unknown. However, studies have shown SCFAs profile in caecum of middle-aged mice is different compared to young or older subjects. In aging mice model, acetate is found significantly increased in middle-aged mice compared to the older subjects [6, 12], butyrate and valerate levels are all higher than that in young mice [6].

Prebiotics are nutrient substances beneficial to the host system through selectively digestion by gut beneficial bacteria. Inulin is one of the well-accepted prebiotics and has shown to increase the abundance of *Bifidobacterium*, resist pathogen colonization and modulate the host immune systems in in vitro gut model systems and humans [23]. The human intervention trials for elderly have shown significant increasing of faecal bifidobacteria after digestion of inulin [24] and fructo-oligosaccharides [25], as well as higher molar ratio of acetate to butyrate and increased cholesterol excretion [24]. In middle-aged mice, feeding inulin supplementation reduces monocyte infiltration into brain and ameliorates age-related microglia activation [6].

Metabolic profiles of gut microbiota can be investigated through in vitro batch fermentation [26], which allows large numbers of substrates and/or faecal samples to be tested quickly [27]. This method has been used in the study of inulin effect [28], healthy modulation by inulin [29] and galacto-oligosaccharide [30, 31]. Our previous studies also showed the in vitro batch fermentation of human feces helps reveal the fermentation characteristics of isomalto-oligosaccharides in colon [32].

Here, we used the batch fermentation model to compare the metabolic responses of faecal microbiota to inulin and soluble starch in midlife and young subjects. Combined with the analysis of microbiota composition, we found the age-related metabolic alterations were associated with the reduction of *Bifidobacterium* in midlife. We refer midlife as the people between 40 to 60 years old [33, 34].

## Results

We investigated the microbial and metabolic features of the faecal microbiota in both young and midlife subjects using in vitro batch fermentation model. Soluble starch, a representative of digestible carbohydrate, and inulin, a representative of non-digestible carbohydrate, were selected to study the metabolic responses. Both the feces and fermented broth samples were processed and subjected to 16S rRNA gene sequencing and SCFAs detection. We also determined gas production in the fermentation model as it is one of the most production metabolites beside SCFAs.

### Difference in the structure of the microbiota community

**Fig. 1:**
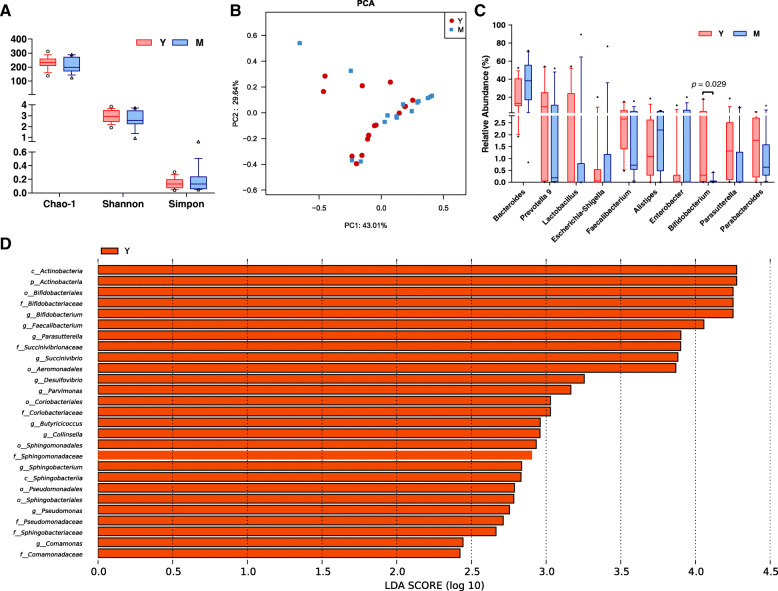
Comparison of diversity and community composition of faecal samples between young and midlife group. **a** Alpha-diversity indices; **b** PCA; **c** relative abundance of top 10 genera; **d** LEfSe

**Fig. 2:**
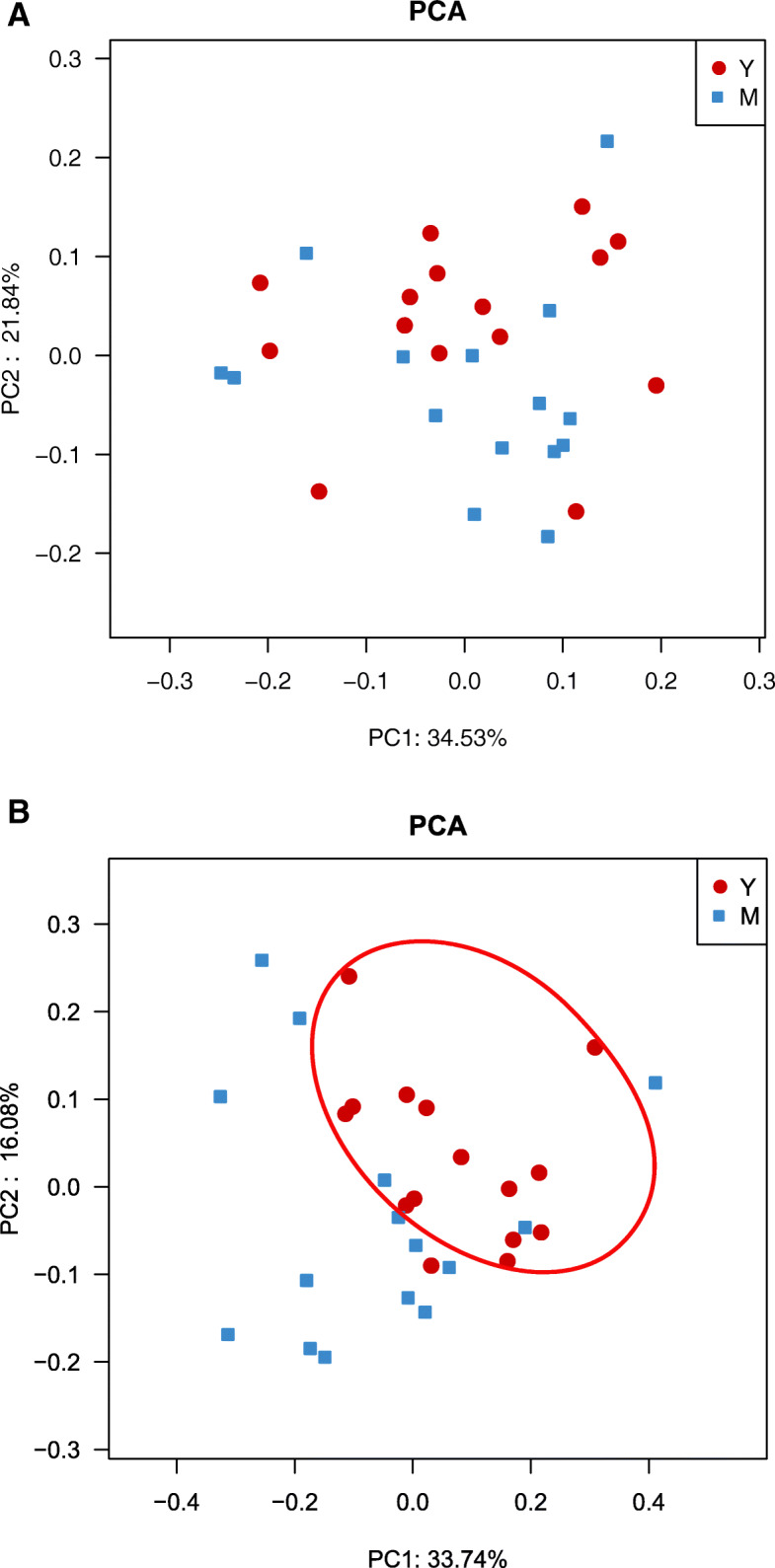
Comparison of the microbial community structures for fermented broth samples between the two age groups. PCA of the microbiota of the broth samples after soluble starch fermentation (**a**), and inulin fermentation (**b**)

Based on the results of the16S rRNA gene sequencing, the alpha-diversity indices, such as Shannon, Simpson and Chao1 index are similar between midlife and young in both faecal and fermented samples (Fig. 1a). We also assessed overall community compositions by drawing bar charts of relative abundance at different taxonomic levels, of which at genus level was shown in supplementary (Supplemental Fig. 1). With the comparison of the relative abundance among top ten genera, *Bifidobacterium* in young group were significantly higher than those in midlife subjects (Fig. 1c). Furthermore, beta-diversity analysis was performed by calculating Bray-Curtis distance matrix to reveal differences between young and midlife group, of which the significance was examined by ANOSIM. Unfortunately, no significant difference was found between the two age groups (Supplemental Fig. 2). Similarly, PCA did not divided the faecal samples into two groups (Fig. 1b). Interestingly, the PCA scattered plots of the inulin broth samples can be obviously clustered on age (Fig. 2b) despite of no difference between age groups in the starch broths (Fig. 2a).

### Impact of environmental factors on microbiota

**Fig. 3:**
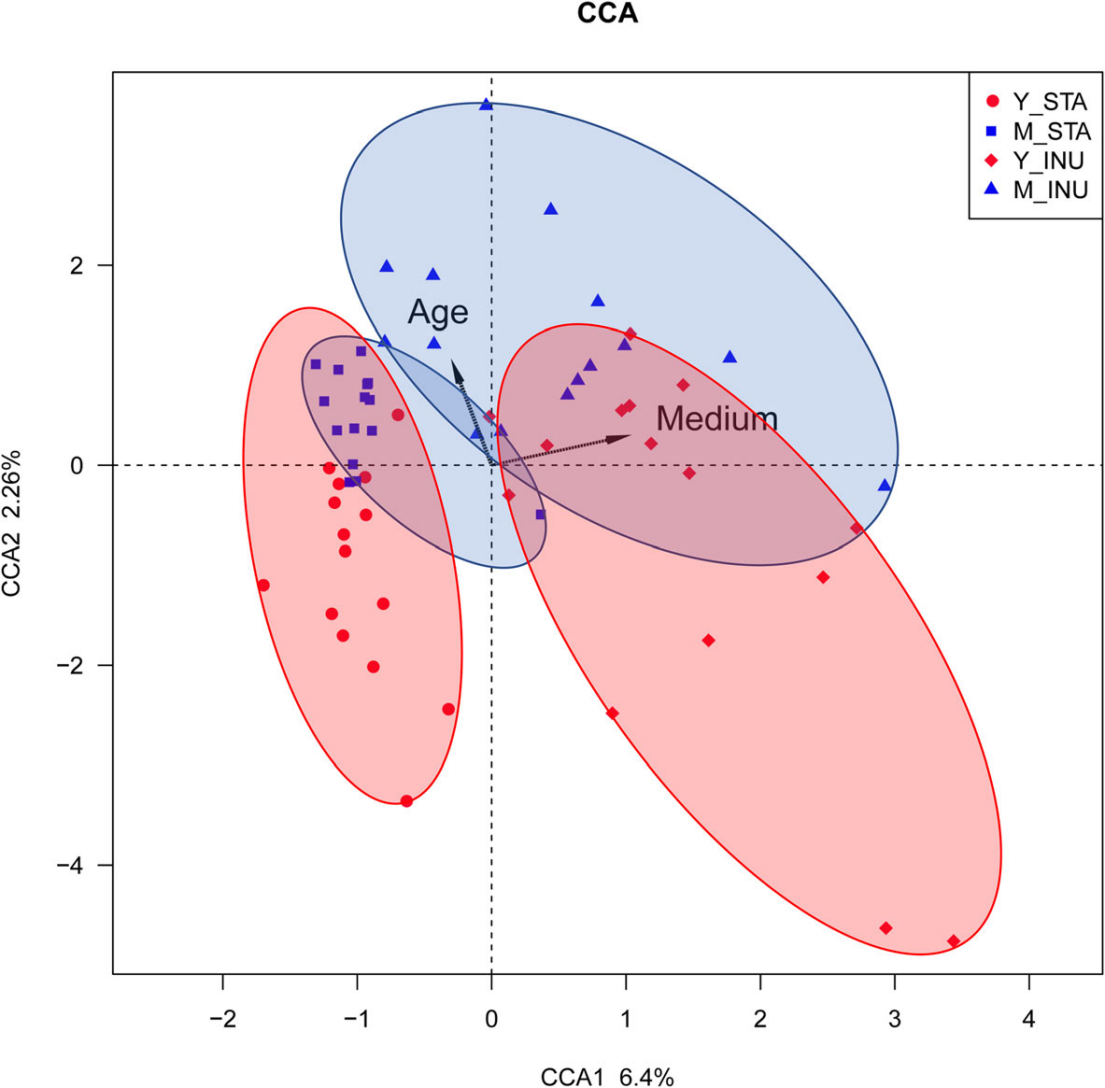
The impact of age and culture medium on microbiota. CCA was used to show the variation in microbiota composition affected by the age groups and the sole carbon hydrates. The samples from midlife (blue) and young (red) group were plotted. The starch fermentation samples were clustered by solid line and inulin fermented samples were clustered by dotted line

Canonical Correspondence Analysis (CCA) was performed to investigate the effects of age and carbon source on the variation in microbiota (Fig. 3). The scatter plot displayed the clustering on age and culture medium. The small percentage of the axis suggests that although age and carbon are not the only sources causing the variation in the system, they are the major ones with more contribution from carbon source than age. This is further supported by the observation that clustering plot shifts in the direction of both medium and age.

### Difference in taxonomic composition

#### Faecal samples

**Fig. 4:**
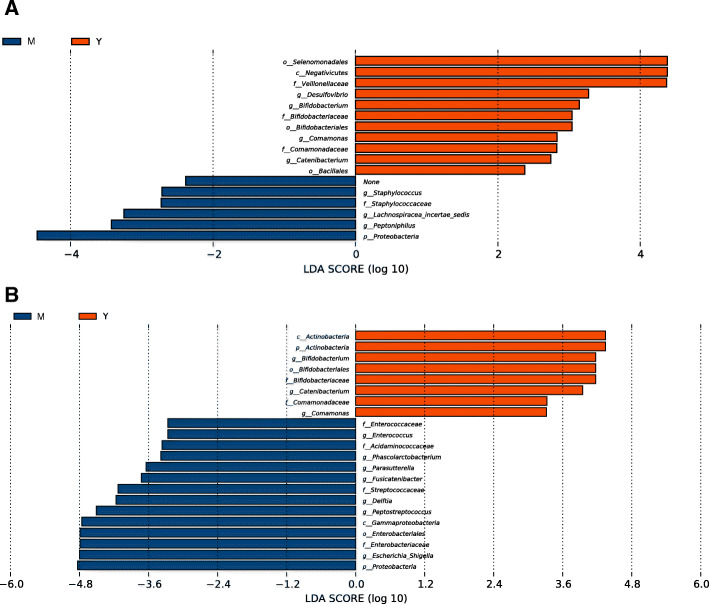
The microbial feature of the microbiota after fermentation. LEfSe of microbiota of the broth samples after soluble starch fermentation (**a**) and inulin fermentation (**b**) were performed. The feature taxa in midlife (M) and young (Y) group were shown according to LDA scores

Linear discriminant analysis (LDA) effect size (LEfSe) was used to identify the age-related bacterial feature in the microbiota before (Fig. 1d) and after fermentation (Fig. 4). As for the faecal samples, LEfSe revealed 27 taxa overrepresent in young group but none in midlife group (Fig. 1d). The taxa involve 11 genera in 4 phyla, suggesting a wide range of changes in relative abundance between youth and midlife. Among the 27 taxa, the top 5 taxa were taxa within phylum Actinobacteria, including Actinobacteria (class), Actinobacteria (phylum), Bifidobacteriales (order), *Bifidobacteriaceae* (family) and *Bifidobacterium* (genus) in turn. Bifidobacteria dominates the infant gut microbiota and reduces with age [35]. Similar to the observation that *Bifidobacterium* disappears in middle-aged mice [12], our data reveal that *Bifidobacterium* is the genus declined most in human midlife. Furthermore, 8 taxa, including *Collinsella* and Coriobacteriales (order) and *Coriobacteriaceae* (family), were observed to decline in midlife. This is also similar to the observation in mice that the relative abundance of both *Bifidobacterium* spp*.* and *Coriobacteriaceae* spp*.* decreases strongly and significantly in middle-aged mice compared to young subjects [12]. Additionally, four genera, including *Faecalibacterium*, *Butyricicoccus, Desulfovibrio* and *Parasutterella* were abundant in young group but absent in midlife group. *Faecalibacterium* and *Butyricicoccus* are the main butyrate products found in the intestine [36, 37]. Thus, the relative abundance of butyrate-producing bacteria differs between young and midlife groups. *Desulfovibrio* is the dominant sulfate-reducing bacteria in the colon and reduces sulfate compounds to hydrogen sulfide (H_2_S) [38], which is one of the main microbiota-derived gases contributing to human colonic homeostasis [39]. *Parasutterella* was the one changed most in phylum Proteobacteria, a result also observed in aging mice [12]. *Parasutterella* is a core component of human gut microbiota and produces various metabolites including aromatic amino acid, bilirubin, purine, and bile acid derivatives [40]. Our results thus suggest the core composition in gut microbiota begins to reduce in midlife.

#### Starch fermented samples

After starch fermentation (Fig. 4a), there were 11 taxa overrepresented in young group, predominated by Selenomonadales (order), Negativicutes (class), *Veillonellaceae* (family), *Desulfovibrio* (genus), *Bifidobacterium* (genus) and its belonging family and order, *Comamonas* (genus) and its belonging family, *Catenibacterium* (genus) and Bacillales (order). In contrast, only 6 taxa were overrepresented in midlife, including Proteobacteria (phylum), *Peptoniphilus* (genus), *Lachnospiracea*-incertae_sedis (genus) and *Staphylococcus* (genus). *Veillonellaceae*, belonging to class Negativicutes and further belonging to order Selenomonadales, is reported to play roles in host carbohydrates metabolism [41]. *Veillonellaceae* and its higher order taxa are the top taxa in starch fermentation indicates their roles seem to be more important for starch degradation compared to *Bifidobacterium*.

#### Inulin fermented samples

In term of the inulin fermentation (Fig. 4b), LEfSe revealed 8 taxa overrepresented in young group compared to midlife group. Five are taxa within phylum Actinobacteria, including *Bifidobacterium* and the remaining 3 taxa are *Catenibacterium* (genus), *Comamonas* (genus) and its belonging family. These taxa are all present in LEfSe results of both feces and starch broth, suggesting they are the key bacteria in young microbiota for saccharolytic process. On the other side, LEfSe identified 14 taxa overrepresented after inulin fermentation in midlife group. The top 5 taxa are Proteobacteria (phylum), *Escherichia-Shigella* (genus)*,* Enterobacteriaceae (family), Enterobacteriales (order), Gama-proteobacteria (class), and the remaining taxa are *Peptostreptococcus* (genus), *Delftia* (genus), *Streptococcaceae* (family), *Fusicatenibacter* (genus), *Parasutterella* (genus), *Phascolarctobacterium* (genus), *Acidaminococcareae* (family), *Enterococcus* (genus), *Enterococcaceae* (family). The shared taxa number within young group after fermentation is 8, contrast to it however, only 1 taxon, Proteobacteria (phylum) is common in midlife group, suggesting that the reduction of bifidobacteria and increase of Proteobacteria can be taxonomic characteristics for midlife microbiota to ferment carbohydrates.

### Detection of Bifidobacterium amount

**Fig. 5:**
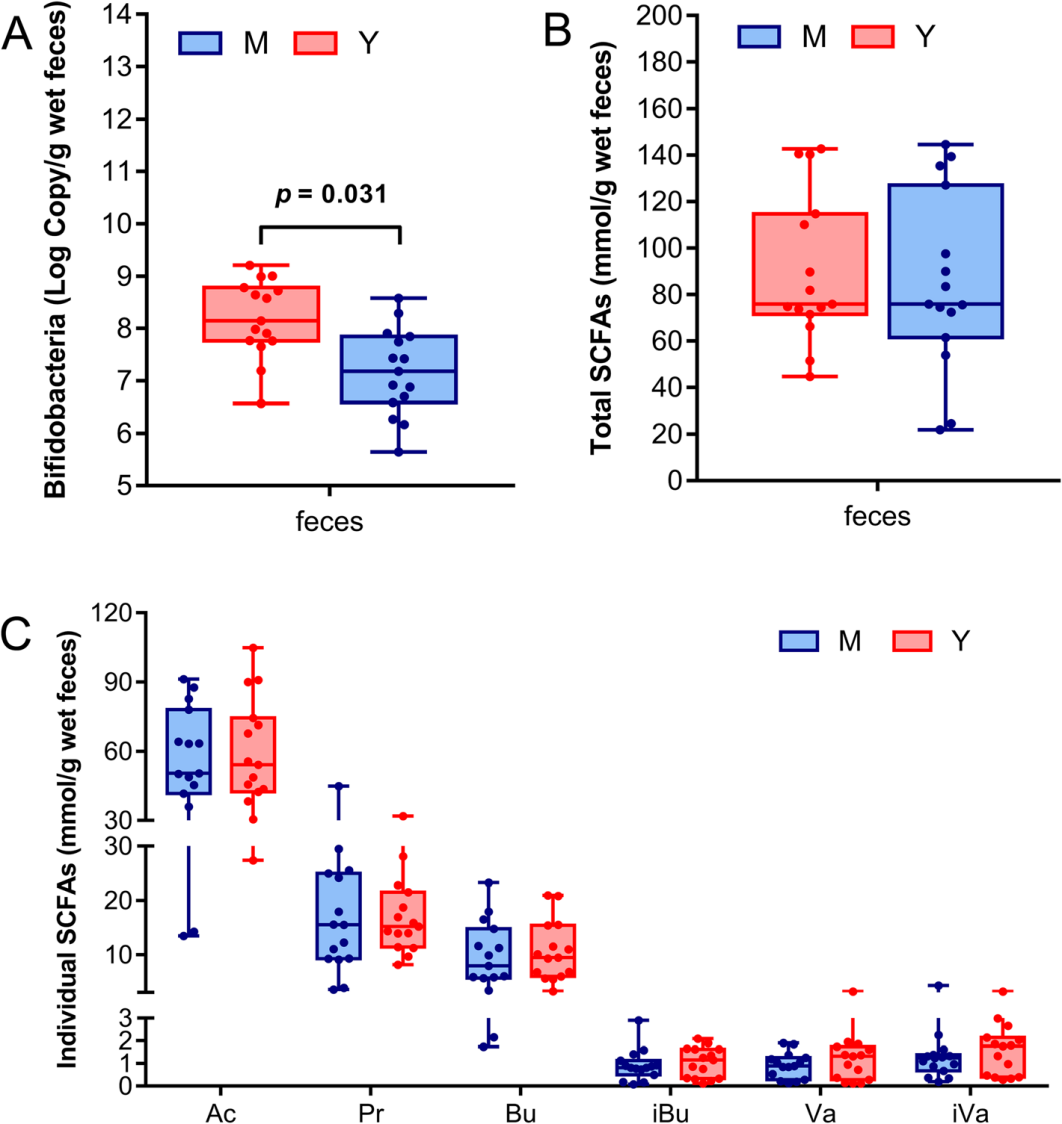
Comparison of the *Bifidobacterium* number and SCFAs in faecal samples. The faecal *Bifidobacterium* numbers of midlife (M) and young (Y) groups were determined by qPCR (**a**). The faecal SCFAs were determined by GC. The total SCFAs (**b**) was the sum of 6 individual acids (**c**). Ac: acetate, Pr: propionate, Bu: butyrate, iBu: isobutyrate, Va: valerate, iVa: isovalerate

**Fig. 6:**
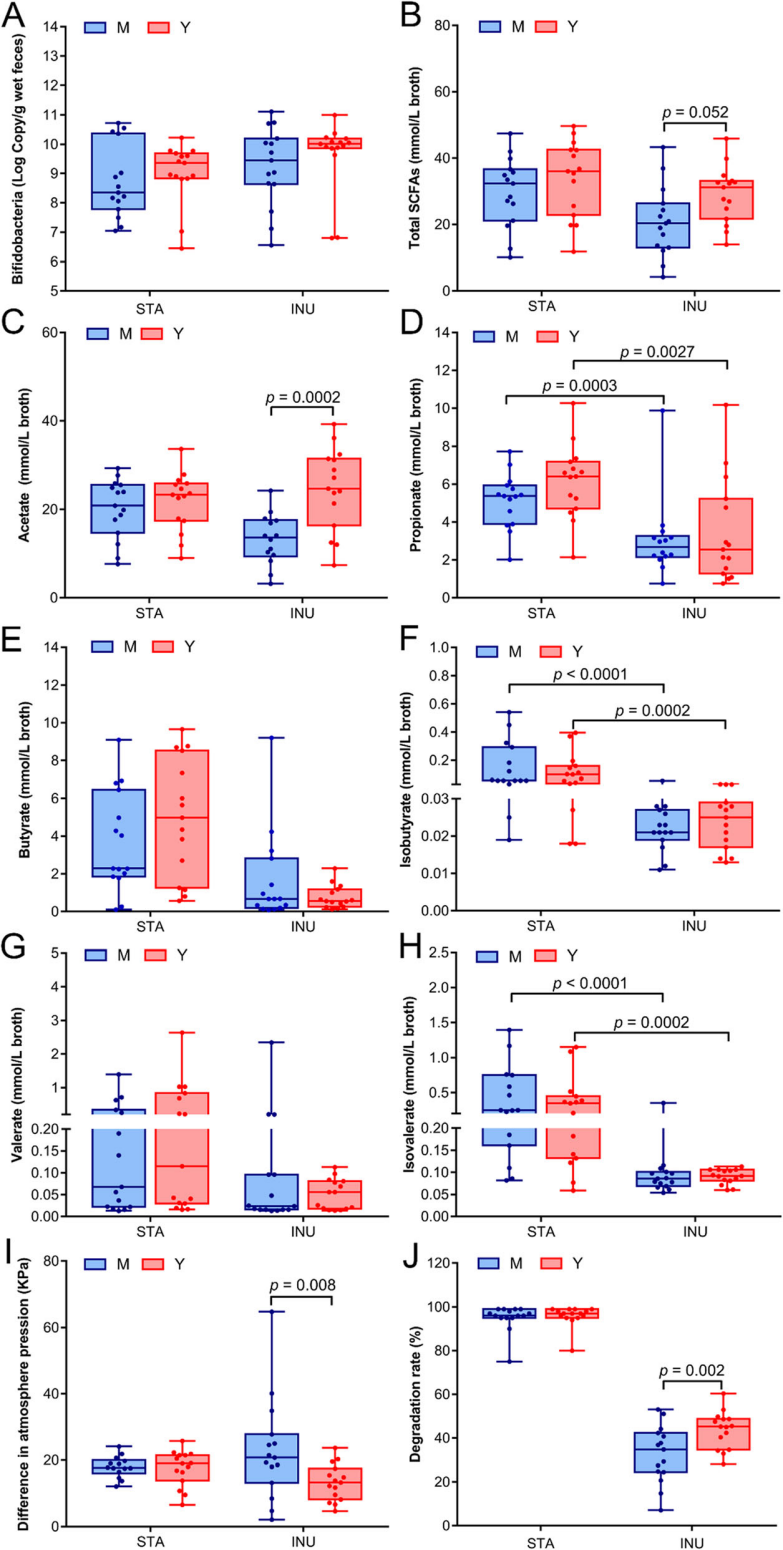
Comparison of the *Bifidobacterium* number and metabolic responses after in vitro batch fermentation. The faecal slurries of midlife (M) and young (Y) groups were subjected to in vitro fermentation of starch (STA) and inulin (INU). The *Bifidobacterium* numbers are determined by qPCR (**a**). The metabolic responses including SCFAs (**b**-**h**), gas productions (**i**) and degradation rate of the sole carbohydrate (**j**) were determined. The SCFAs were determined by GC. The total SCFAs (**b**) is the sum of 6 individual acids including acetate (**c**), propionate (**d**), butyrate (**e**), isobutyrate (**f**), valerate (**g**) and isovalerate (**h**). The gas production after fermentation was measured by the air pressure difference of the vial (**i**). The degradations of starch and inulin after fermentation were detected by TLC (**j**)

To confirm the LEfSe results regarding *Bifidobacterium*, qPCR was performed for the faecal samples (Fig. 5a) and the fermented samples (Fig. 6a). The abundances of *Bifidobacterium* in midlife group were overall lower than that in young group, particularly in the faecal samples (*p* = 0.031) (Fig. 5a).

### Difference in metabolic response

SCFAs in both feces and fermented broths were measured. As shown in Fig. 5c, most individual SCFAs in the feces in midlife group were lower than those in young group, except for propionate, but without significant difference. After starch fermentation, the most individual SCFAs in midlife were lower than those in young group (Fig. 6c-h), which is similar to the case in feces. However, after inulin fermentation, the reduction of acetate in midlife (Fig. 6c) was significant (*p* = 0.0002) compared to young group despite of similar level of butyrate and propionate in the two age groups. A similar result has been observed in aging mice that butyrate and propionate do not differ between two age groups, but acetate is significantly lower in old mice [12]. It is worth pointing out that the total SCFAs in midlife broth of inulin declines pronouncedly (*p* = 0.052). In addition, inulin fermentation also caused significant gas production increase in midlife group (*p* = 0.008) (Fig. 6i), and inulin degradation decrease (*p* = 0.002) compared to young group (Fig. 6j), a result comparable to the decline of saccharolytic capability in aging mice [11].

### Correlation between the metabolites and the genera

**Fig. 7:**
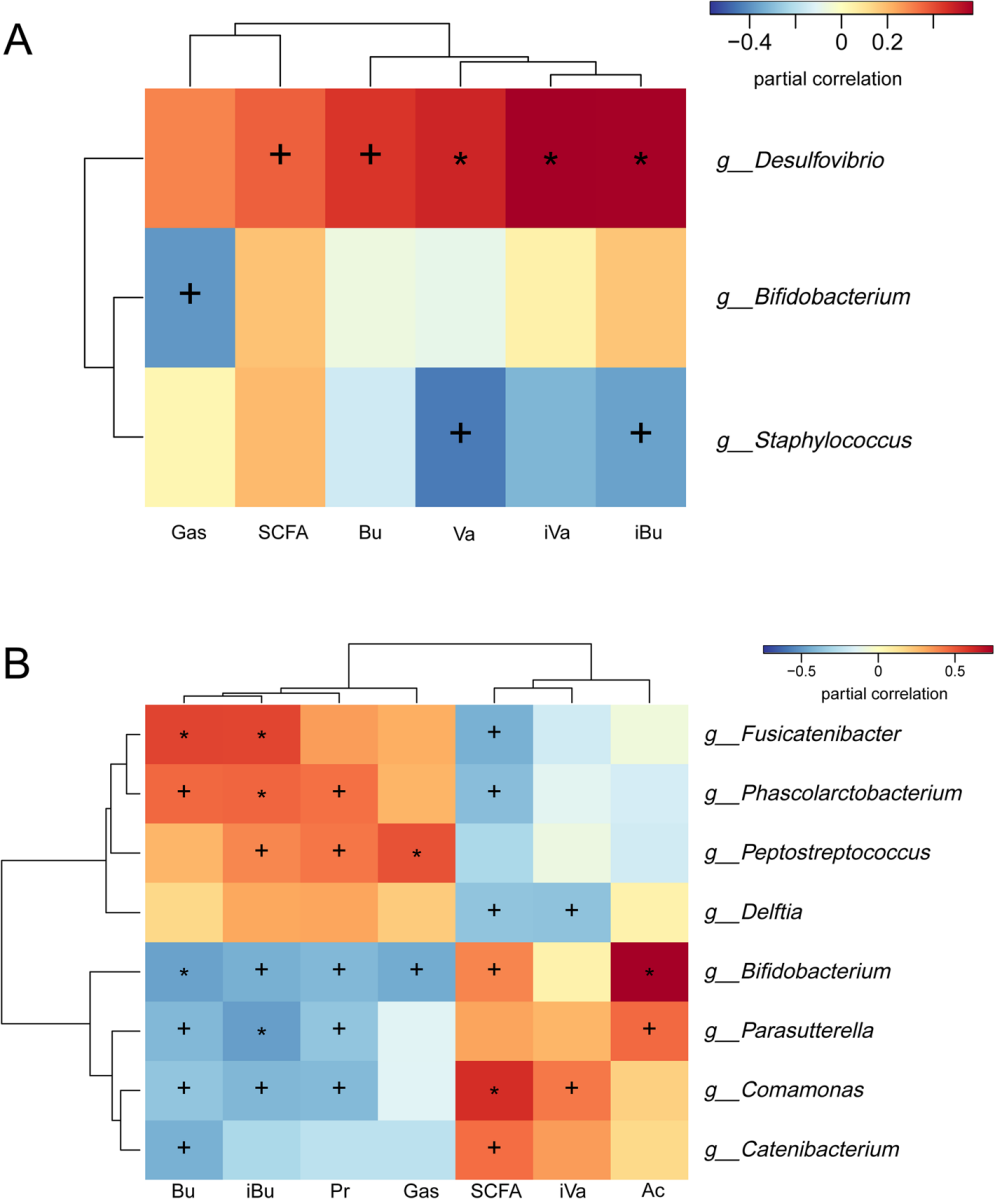
The correlation of metabolites and fermented microbiota composition. Spearman’s correlation coefficient analysis was performed to reveal the relationship between the metabolites and fermented microbiota of starch (**a**) and inulin (**b**). The calculated coefficients are shown in heatmap. Ac: acetate, Pr: propionate, Bu: butyrate, iBu: isobutyrate, Va: valerate, iVa: isovalerate, +: *p* < 0.05, *: *p* < 0.01

To understand the contribution of genera to the altered metabolites, Spearman’s correlation coefficient was used to analyze the association between metabolites concentration and genus abundance. As for the starch fermentation, 3 genera, including *Bifidobacterium*, *Desulfovibrio* and *Staphylococcus*, showed correlation with six metabolic parameters (Fig. 7a). As for inulin fermentation, 8 genera included *Fusicatenibacter*, *Phascolarctobacterium*, *Peptostreptococcus*, *Delftia*, *Bifidobacterium*, *Parasutterella*, *Comamonas* and *Catenibacterium* were involved and correlated with seven metabolic parameters (Fig. 7b).

As shown in Fig. 7b, only two genera, *Bifidobacterium* and *Parasutterella*, showed significant correlation with acetate production, which were both positive. Upon the heatmap, *Bifidobacterium* had stronger correlation with acetate production than *Parasutterella*, suggesting the reduced acetate in midlife group might be responsible for the less abundance of *Bifidobacterium*, a powerful acetate producer. On the other hand, *Peptostreptococcus* and *Bifidobacterium* were the only genera shown significant correlation with gas production, which was positive and negative, respectively. This indicated the increased gas production in midlife group also seemed to be due to the absence of *Bifidobacterium*, an inhibitor of gas production.

## Discussion

We studied the difference of faecal microbiota between midlife and young subjects by the investigation of both the community structures and the metabolic responses before and after in vitro fermentation. Our results suggested multi-aspect changes in midlife microbiota and its functions.

We observed a significant reduction of *Bifidobacterium* in both midlife faecal and fermented microbiota. The community composition analysis revealed that *Bifidobacterium* was significantly reduced in midlife faecal microbiota compared to young subjects, which was confirmed by qPCR. Furthermore, LEfSe showed that young group had more abundance *Bifidobacterium* than midlife group not only in feces but also in the fermented samples both of soluble starch and inulin. *Bifidobacterium* is thought to play pivotal roles in maintaining human health [42, 43], and its numbers decline is one of the most marked changes in the elderly gut [44]. Thus, the reduced *Bifidobacterium* seems to be the age-related feature in midlife microbiota. In addition, LEfSe also revealed that the core composition in gut microbiota begins to reduce in midlife feces, which involved 27 taxa, such as genus *Faecalibacterium* which also associates with the grip strength in elderly [45]. On the other hand, an age-related change in the microbial community structure of midlife was revealed by PCA after in vitro fermentation of inulin and the subsequent analysis of CCA, despite of similarity in the diversity analysis, including alpha-diversity indies and the distance matrix calculating-based beta-diversity analysis, which agrees with the previous reports that the faecal microbiota structures of 20 to 60 years old are in a stable stage in this aspect [46, 47]. Hence, we conclude that microbial alterations associated with aging also occur in midlife, rather than the previous thought to begin in elderly. This is consistent with previous report of age-related functional characteristics in metagenomes that midlife with age 38–43 differentiates from young and elderly and constitutes a good watershed in data clustering between age groups [48].

The metabolic responses to inulin were significantly different between midlife and young group. After inulin fermentation, midlife group had lower acetate and higher gas production compared to young group, as well as lower inulin degradation rate. Based on the Spearman’s correlation coefficient analysis, *Bifidobacterium* was positively correlated with acetate production and negatively correlated with gas production. Thus, the absence of *Bifidobacterium* in midlife seemed to contribute to both the significantly increased gas production and the significantly reduced acetate after inulin fermentation. This is supported by the recent reports that *Bifidobacterium* is lack of hydrogenase genes in its genome [49] and acetate is its main metabolite [50]. In human intervention trial of resistant starch, some volunteers were found as “non-responders” with > 60% of unfermented starch remaining in their stools while others with < 4% [51]. The difference in the initial composition of individual’s gut microbiota may be the cause [52, 53]. Therefore, the reduction of *Bifidobacterium* in midlife feces seems to be responsible for the significant drop in metabolic responses to inulin for midlife microbiota.

Bifidobacteria is reported to own the capacity to ferment a variety of carbohydrate and fiber compounds [54]. The saccharolytic genes were found to decrease in elderly gut microbiota [21] and older age mice [11]. However, the attenuated saccharolytic capacity only found in the fermentation of inulin but not starch, suggesting that the microbial changes in midlife associated with aging are still in initial stage, leading to a partial functional decline.

In this study, acetate was significantly reduced in midlife after inulin fermentation compared to young group. SCFAs have a number of potential roles in modulating metabolic health [19]. Acetate is most productive acid among SCFAs and appears to stimulate leptin secretion in adipocytes, involving energy balance and appetite [16], conditioning immune cell in response to protect against T2D [55] and regulating blood pressure [56, 57]. And the impaired acetate production might be associated with weight gain in midlife [58]. Considering that approximately 44% acetate in plasma is microbiota-derived [59], the reduced production of acetate by microbiota might be associated with the physiological changes in midlife which leading obesity [7], T2D [8] and cardiovascular disease [10].

Interestingly, contrast to significantly reduced inulin degradation and acetate production, the reduction in number of *Bifidobacterium* is not significant after inulin fermentation. Concerning its diversity of inulin catabolism [60] and its niche- and strain-specific acetate production [61], the loss of certain *Bifidobacterium* species or strains that produce large amounts of acetate might be the reason. This is supported by a recent study that changes in the composition of *Bifidobacterium* species occur with ageing [35]. Addition to the roles of acetate in the healthy problem of midlife, such as obesity and T2D, it is reported that bifidobacteria-produced acetate improves intestinal defense mediated by epithelial cells and thereby protects the host against lethal infections [62]. Therefore, further study is needed to clarify the species of *Bifidobacterium* reduced in midlife.

## Conclusions

The gut microbiota compositions and the metabolic responses of the microbiota for midlife and young subjects were investigated by using in vitro batch fermentation of starch and inulin. *Bifidobacterium* reduction is identified as the feature of midlife microbiota and results in deficient saccharolytic capacity leading to a significant decline of acetate production in inulin fermentation, which seems to associate with the diseases prevalent in midlife. Overall, our results indicate the advantage of in vitro batch fermentation model in both microbiota and its metabolites analysis.

## Methods

### Subjects

Healthy volunteers aged 20–39 and 40–60 years old were recruited, 15 individuals in each group. The average age of the youth group (20–39 years old) was 26.6 ± 1.6 years old and of the mid-life group (40–60 years old) was 50.9 ± 1.6 years old. Exclusion criteria included: 1) Any gastrointestinal disease such as IBS and IBD; 2) Presence of severe diabetes and unstable thyroid disease; 3) Pregnant or lactating women; 4) Taking probiotics, prebiotics and antibiotics agents within 4 weeks prior to the faecal samples collection. All volunteers were provided with informed, written consent and instructed to collect the feces.

### Fermentation media

Batch fermentation was conducted as described previously [63]. In brief, basic growth medium (YCFA) contained the following (g/L): tryptone 10; yeast extract 2.5; L-cysteine 1; NaCl 0.9; CaCl_2_·6H_2_O 0.09; KH_2_PO_4_ 0.45; K_2_HPO_4_ 0.45; MgSO_4_·7H2O 0.09; vitamin I 200; hemin solution 2 ml. Vitamin I solution contained the following compositions (mg/ml): vitamin B8 0.05; vitamin B12 0.05; acid 4-aminobenzoïque 0.15; vitamin B9 0.25; pyridoxamine 0.75. The hemin concentration is 1 mg/ml in 1 M sodium hydroxide. Inulin (Fengning Ping An High-Tech, China, VILOF®-NanoIN) or soluble starch (Sigma-Aldrich, V900508) was added (8 g/L) as the sole carbon source. After added resazurin (0.1 mg/L), the indicator of anaerobic condition, the medium was adjusted to pH 6.5 and dispensed 5 ml into a 10 ml bottle with flushing N_2_ before autoclave.

### Batch fermentation

Fresh faecal samples (0.8 g) were homogenized with 8 mL of 0.1 M anaerobic phosphate-buffered saline (pH 7.0) using an automatic faecal homogenizer (Halo Biotechnology Co. LTD., China) to make 10% (w/v) slurries as soon as the faecal samples arrived the laboratory. And 0.5 ml of the faecal slurry was inoculated to 5 ml growth media and subjected to anaerobic fermentation at 37 °C.

After 24 h of fermentation, the air pressure difference of the serum bottle was measured. Subsequently, the broth was aliquoted and centrifuged. The supernatant and precipitation of the broth were stored at − 30 °C for the further metabolic analysis and DNA extraction.

### Determination of gas production

The gas production capacity was determined by the air pressure difference of the serum bottle using digital manometer (Dongguan Xintai Instrument Co., Ltd., China, HT1895).

### Thin-layer chromatography (TLC)

Degradation of starch and inulin was detected by TLC analysis described in the previous study [32]. The densities of the oligosaccharide spots in the TLC images were quantified by Quantity One (Bio-Rad, U.S.). The consumed amounts of oligosaccharides by bacteria were calculated by the difference of the densities between the medium and the broth. The degradation rate of oligosaccharide was the percentage of the amount consumed versus its original amount in the medium.

### SCFAs determination

SCFAs were determined via gas chromatography as described previously [64]. Briefly, 1 mL of broth or faecal slurry was acidified by 0.2 mL of 25% (w/v) metaphosphoric acid and centrifuged at 14,000 g for 2 min. The filtered supernatant was analyzed on GC-2010 Plus gas chromatograph (Shimadzu, Japan) equipped with an H_2_ flame ionization detector. A DB-FFAP column (0.32 mm × 30 m × 0.5 μm) (Agilent Technologies, US) was used to separate SCFAs. Crotonic acid (trans-2-butenoic acid) was used as an internal standard.

### DNA extraction

Bacterial genomic DNA was extracted using QIAamp DNA Stool Mini Kit according to the manufacturer’s instructions (Qiagen, Germany). The concentration of extracted DNA was determined by NanoDrop ND-2000 (NanoDrop Technologies, U.S.), and confirmed by 1.0% agar gel electrophoresis [65].

### 16S rRNA gene sequencing

Bacterial 16S rRNA genes of V3-V4 were amplified using barcoded primers 341F (5′-CCTAYGGGRBGCASCAG-3′) and 806R (5′-GGACTACNNGGGTATCTAAT-3′). Amplicons were extracted from 2% agarose gels and purified using AxyPrep DNA Gel Extraction Kit (Axygen Biosciences, Union City, CA, U.S.) according to the manufacturer’s instructions and quantified using QuantiFluor™ -ST (Promega, U.S.). Purified amplicons were pooled in equimolar and paired-end sequenced (2 × 250) on Illumina MiSeq platform according to the standard protocols, performed by Lizhen Biopharmaceutical technology co., Ltd., Hangzhou, China.

Sequences were identified by barcodes using Quantitative Insights in Microbial Ecology (QIIME) 1.8.0 pipeline. Low quality sequences were removed for further analysis. Sequences were clustered into the operational taxonomic units (OTUs) with 97% similarity cutoff. The OTUs were assigned to taxa using the Greengenes database 2 (Release 13.8). A phylogenetic tree of representative sequences was built. The within-community diversity (alpha-diversity) indies, such as Chao1, Simpson and Shannon, were calculated by the R “phyloseq” package. Beta-diversity was estimated by calculating Bray-Curtis distance matrix by R package vegan and ggplot2 and statistically examined by ANOSIM. PCA was performed to explore variance in microbiota composition. Age and carbohydrate source for the culture were treated as environmental factors. Their impact on microbiota was evaluated by CCA. Age dependent features were identified by using LEfSe. Heatmaps were built to show Spearman’s correlation coefficient between metabolites and microbiota composition at genus level. The input consisted of the OTUs with relative abundance more than 0.1% at least in one sample. The raw reads were deposited into the NCBI Sequence Read Archive (SRA) database (Accession Number: SRP187258).

### Quantitative PCR (qPCR)

The number of *Bifidobacterium* in the feces and broth samples were assessed by qPCR in CFX96 TM Real-time PCR Detection System (Bio-Rad, U.S.) with specific primers Bifi601F: 5′-GGGTGGTAATGCCGGATG and Bifi601R: 5′-TAAGCCATGGACTTTCACACC-3′. The qPCR was performed as described previously [32, 66].

### Statistical analysis

Statistical analyses were performed using Mann-Whitney test or multiple t tests using Holm-Sidak method by GraphPad Prism 7.00 software. *p*-values < 0.05 were considered statistically significant.

## Supplementary Information

**Additional file 1: Supplemental Fig. 1**. Relative abundance of genus taxonomic level within individual faecal samples.

**Additional file 2: Supplemental Fig. 2**. ANOSIM test of Bray-Curtis distance matrix calculation. (A) Faecal samples, (B) broth samples of starch fermentation, (C) broth samples of inulin fermentation.

## Data Availability

The 16S rRNA gene amplicon sequence dataset has been uploaded to the NCBI Sequence Read Archive (SRA) under accession number SRP187258 (https://www.ncbi.nlm.nih.gov/sra/?term=SRP187258). Other datasets analyzed during the current study are available from the corresponding author on reasonable request.

## Abbreviations

CCA: Canonical Correspondence Analysis
LEfSe: Linear discriminant analysis (LDA) effect size
PCA: Principal Component Analysis
qPCR: quantitative PCR
SCFAs: short chain fatty acids
T2D: type 2 diabetes
TLC: thin-layer chromatography
