# Supplementary figures and images for: Age-related changes of microbiota in midlife associated with reduced saccharolytic potential: an in vitro study
Source: BMC Microbiol. 2021 Feb 15;21:47. doi: 10.1186/s12866-021-02103-7 (PMC7885556; doi:10.1186/s12866-021-02103-7)

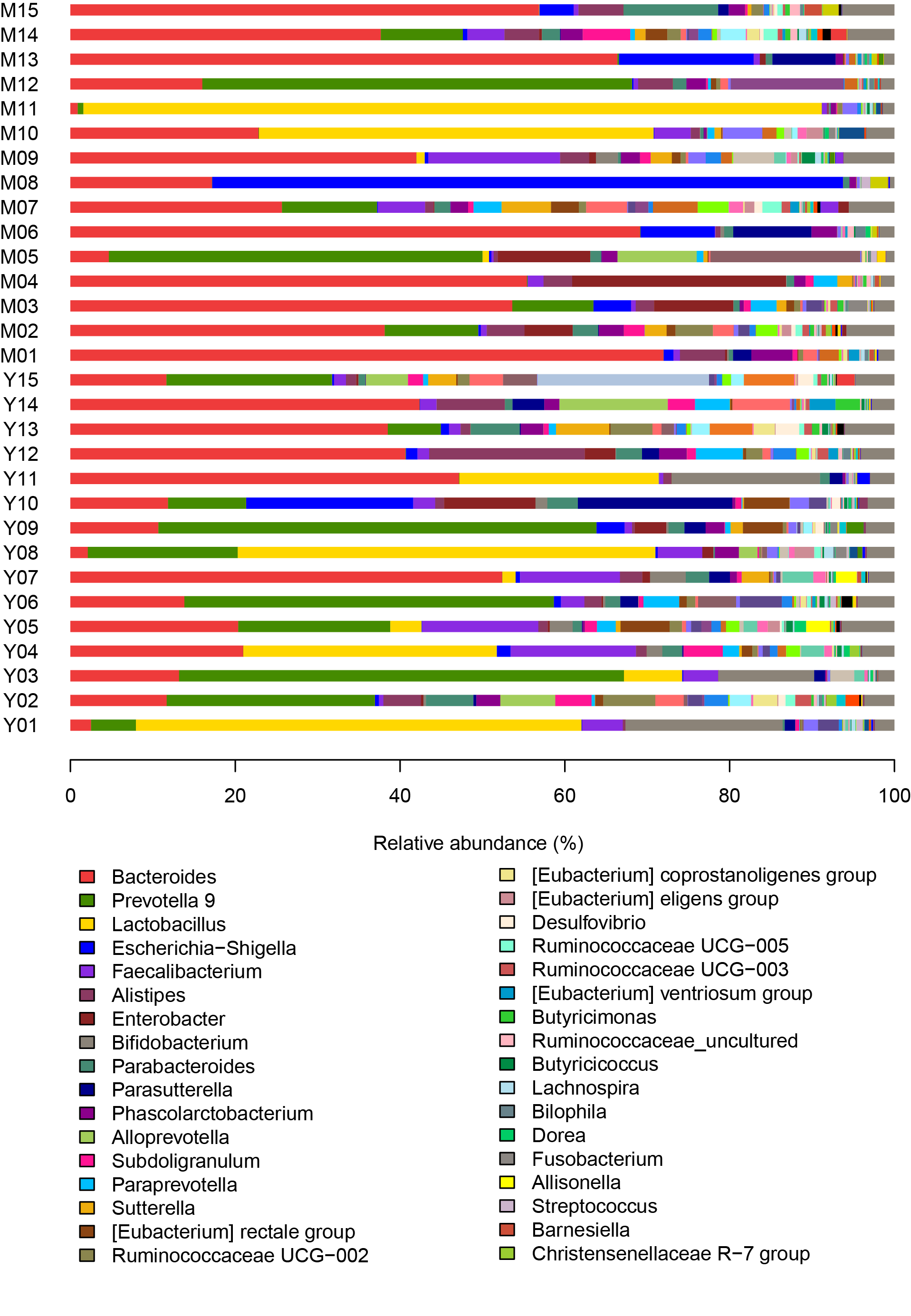

Supplement: Supplementary file 1 — Additional file 1: Supplemental Fig. 1. Relative abundance of genus taxonomic level within individual faecal samples. [file 12866_2021_2103_MOESM1_ESM.png]

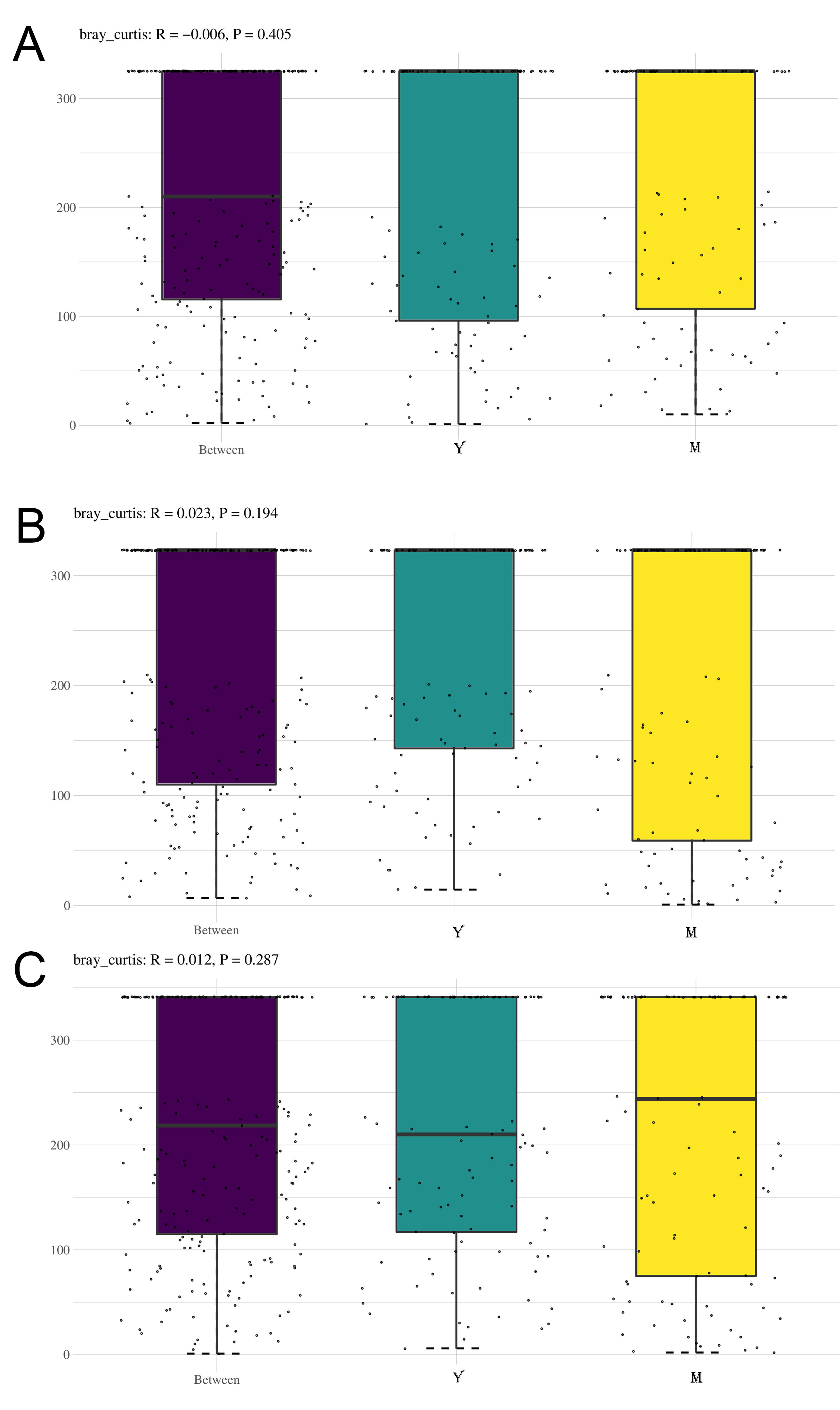

Supplement: Supplementary file 2 — Additional file 2: Supplemental Fig. 2. ANOSIM test of Bray-Curtis distance matrix calculation. (A) Faecal samples, (B) broth samples of starch fermentation, (C) broth samples of inulin fermentation. [file 12866_2021_2103_MOESM2_ESM.jpg]
